# Sequences of cognitive decline in typical Alzheimer's disease and posterior cortical atrophy estimated using a novel event‐based model of disease progression

**DOI:** 10.1002/alz.12083

**Published:** 2020-06-02

**Authors:** Nicholas C. Firth, Silvia Primativo, Emilie Brotherhood, Alexandra L. Young, Keir X.X. Yong, Sebastian J. Crutch, Daniel C. Alexander, Neil P. Oxtoby

**Affiliations:** ^1^ Centre for Medical Image Computing Department of Computer Science UCL London WC1E 6BT UK; ^2^ Dementia Research Centre UCL Queen Square Institute of Neurology UCL London WC1N 3BG UK; ^3^ Department of Human Science LUMSA University Rome Italy; ^4^ Department of Neuroimaging Institute of Psychiatry, Psychology and Neuroscience King's College, London, UK; ^5^ Clinical Imaging Research Centre National University of Singapore Singapore

**Keywords:** Alzheimer's disease, ceiling, cognitive decline, dementia, disease progression model, effect, floor, kernel density estimate, non‐Gaussian, nonparametric mixture model, posterior cortical atrophy

## Abstract

**Introduction:**

This work aims to characterize the sequence in which cognitive deficits appear in two dementia syndromes.

**Methods:**

Event‐based modeling estimated fine‐grained sequences of cognitive decline in clinically‐diagnosed posterior cortical atrophy (PCA) (n=94) and typical Alzheimer's disease (tAD) (n=61) at the UCL Dementia Research Centre. Our neuropsychological battery assessed memory, vision, arithmetic, and general cognition. We adapted the event‐based model to handle highly non‐Gaussian data such as cognitive test scores where ceiling/floor effects are common.

**Results:**

Experiments revealed differences and similarities in the fine‐grained ordering of cognitive decline in PCA (vision first) and tAD (memory first). Simulation experiments reveal that our new model equals or exceeds performance of the classic event‐based model, especially for highly non‐Gaussian data.

**Discussion:**

Our model recovered realistic, phenotypical progression signatures that may be applied in dementia clinical trials for enrichment, and as a data‐driven composite cognitive end‐point.

## 1 INTRODUCTION

Alzheimer's disease (AD) is the leading contributor to the global dementia epidemic. With no disease‐modifying therapeutic currently available and over 99% of clinical trials concluding without evidence of efficacy for their putative therapy,^1^, ^2^, ^3^, ^4^, ^5^ there is an immediate urgency to improve our understanding of AD, and dementia in general, to inform these efforts.

It is estimated that measurable changes in biomarkers can occur decades before a dementia diagnosis is made, such as amyloid abnormality in typical (memory‐led) AD.^6^ This makes dementia particularly challenging to study because it is near‐impossible to discern precisely when a given change occurs from a cohort of diagnosed or prodromal individuals, without following them for a prohibitively long period of time.

The event‐based model^7^, ^8^ is a computational algorithm specifically designed to meet this challenge, uniquely using only a cross‐sectional sample such as the baseline visit in a study. The event‐based model estimates the ordered sequence of abnormality in a set of biomarkers by combining severity information across biomarkers and individuals, without reference to a given individual's clinical status. It has been applied in both sporadic and familial AD,^8^, ^9^, ^10^, ^11^ Huntington's disease,^12^, ^13^ and progressive multiple sclerosis.^14^ Recently, the event‐based model idea has been extended to a new algorithm for finding data‐driven subtypes of disease,^15^ demonstrated in AD and frontotemporal dementia. These investigations, and others,^16^ focused largely on neuroimaging biomarkers, made possible by the increasing availability of large datasets such as from the Alzheimer's Disease Neuroimaging Initiative and the Dominantly Inherited Alzheimer Network.

In contrast to neuroimaging, most analyses of complex cognitive datasets have relied on traditional statistical approaches rather than data‐driven methods. Methods of detecting cognitive change are important both for improving disease characterization and prognosis in affected individuals, and for detecting and predicting change in individuals who are asymptomatic and at‐risk (of sporadic disease) or presymptomatic (have a familial/genetic disease).^17^ Optimizing methods for analyzing cognitive change is especially important in the context of clinical trials, because cognitive and functional outcomes are currently the only accepted means for proving efficacy of a drug. This is relevant to both symptomatic trials and secondary prevention trials, because neuropsychological tests may be sensitive to dementia between 10 and 17 years before diagnosis.^18^

Evaluation of longitudinal change within and across cognitive domains presents a number of specific challenges. First, performance across cognitive tasks is not independent. General factors (eg, disease severity) and collateral deficits (eg, visuoperceptual problems limiting performance on a visual memory test) can influence testing across domains. Second, cognitive profiles across tasks are often described qualitatively because test properties and normative samples differ across tasks. Third, the psychometric shape of tests can differ markedly. Tests involving graded difficulty yield relatively linear score distributions among healthy control participants, whereas other tests may yield skewed, highly non‐Gaussian score distributions owing to an excess of very‐easy or very‐difficult items. These properties influence the likelihood of clinical populations showing ceiling or floor effects at any given point in their disease progression. Fourth, practice effects mask longitudinal change, for example, test familiarity and/or reduced anxiety may conceal evidence of cognitive instability or decline.^19^

We are motivated to understand disease progression in Alzheimer's and dementia, with a focus on impact for interventional trials and in the clinic. International Working Group criteria now include explicit definition of atypical forms of AD,^20^, ^21^ of which posterior cortical atrophy (PCA) is acknowledged to be one of the most common.^22^, ^23^ It is of fundamental importance to understand disease progression in both typical and atypical AD if the field is to advance. PCA is a clinico‐radiological syndrome characterized by progressive decline in visual processing and other posterior cognitive functions, relatively intact memory and language in the early stages, and atrophy of posterior brain regions.^20^, ^21^ PCA is most commonly caused by AD, with greater presence of amyloid plaque and/or neurofibrillary tangles in the posterior cortices than in individuals having the more‐typical, amnestic presentation.^24^, ^25^ Detailed longitudinal studies of cognitive change in PCA are understandably rare.^26^

Here we aimed to estimate the sequence of cognitive decline in typical AD (tAD) and PCA. This necessitated development of a new event‐based model designed specifically to handle highly non‐Gaussian input data for data‐driven assessment of cognitive decline. We validated our new model in simulation experiments (documented in Supplementary Material) before applying to patient data from our cohorts.

**RESEARCH IN CONTEXT**

1. Systematic review: We reviewed the literature using PubMed and found no data‐driven estimation of cognitive decline in posterior cortical atrophy.
2. Interpretation: Our data‐driven results reveal new understanding into the sequence of detectable cognitive decline in posterior cortical atrophy and tAD, including the first direct comparison. Our novel method produces a fine‐grained prognostic tool useful for staging individuals based on their cognitive profile, and for predicting subsequent decline.
3. Future directions: We foresee multiple avenues for future work. (1) Validation of our model using larger datasets in tAD. (2) Application of these models to clinical trials in tAD and posterior cortical atrophy (enrichment; and data‐driven composite cognitive end‐points). (3) Wider application of our new method in other neurodegenerative diseases and beyond neuropsychological test scores, including data‐driven subtyping of diseases. (4) Working toward making KDE mixture modeling a more general statistical methodology.

## 2 MATERIALS AND METHODS

### 2.1 Participants in the two dementia cohorts

Patients with a clinical diagnosis of PCA (n=94) or tAD (n=61) were recruited between October 2005 and June 2016 at the UCL Dementia Research Centre in London. Healthy controls (HC) (n=23) were sampled from the Young Onset Alzheimer's Disease study (Table 1) at the same center. Our control group is younger than both patient groups (Mann‐Whitney *U* test, P<.05). Patients were recruited via attendance at the Cognitive Disorder Clinic at the National Hospital for Neurology and Neurosurgery, or were recruited by individual referral from neurologists to whom they expressed interest in taking part in observational research. All PCA patients met both Tang‐Wai et al.^24^ and Mendez et al.^27^ criteria based on available information at baseline and expert retrospective clinical review by consultant neurologists and neuropsychologists with expertise in cognitive neurology. PCA participants were excluded if they also met criteria for another neurodegenerative syndrome, thus our PCA group fulfils consensus criteria for PCA‐pure.^23^ Patients with PCA and patients with tAD fulfilled research criteria for probable AD.^28^, ^29^ Examples of excluded syndromes include dementia with Lewy bodies, corticobasal degeneration, and prion disease; with clinical features such as visual hallucinations, pyramidal signs, reduplicative phenomena, parkinsonism, dystonia, myoclonus, and ataxia. Additionally, tAD patients did not fulfil clinical criteria for logopenic variant of primary progressive aphasia^30^ nor frontal variant AD.^20^ Event‐based models (see Section 2.3.1) were fit to baseline data for PCA and tAD separately. Follow‐up visits were used to assess longitudinal self‐consistency of each model: the mean ± std number of visits per patient is 3.1±1.1 (range 2–6) for PCA and 2.2±0.4 (range 2–3) for tAD. We investigated controlling for visual acuity to avoid confounding visual cognition, but we found it impossible to separate acuity from cognition in our PCA patients — even on acuity tests intended to be robust to deficits in visual processing. Information on patient medication was not routinely collected in our cohorts.

**TABLE 1:** Demographics of participants

	PCA (n=94)	tAD (n=61)	HC (n=23)
Age (years)	64.2 (7.9)	65.8 (7.7)	60.2 (5.7)
Gender (m:f)	35:59	38:23	11:12
MMSE, mean (std)	21.4 (5.1)	19.6 (4.9)	29.5 (0.7)
N visits, mean (std)	2.5 (1.3)	1.6 (0.6)	1.8 (0.4)

Abbreviations: HC, healthy control; MMSE, Mini‐Mental State Examination; PCA, posterior cortical atrophy; tAD, typical Alzheimer's disease.

Data collection was approved by the National Research Ethics Service Committee London (UK National Health Service Health Research Authority). All participants provided written informed consent according to guidelines established by the Declaration of Helsinki.

### 2.2 Neuropsychological tests

We employed a battery of tests that are routinely used in the clinic^31^, ^32^, ^33^, ^34^, ^35^ and in pharmacological^36^ and non‐pharmacological^37^ clinical trials involving PCA. We used the same battery of neuropsychological tests on each cohort, which allows direct comparison of cognitive decline across the two dementia syndromes. The battery includes assessments of episodic and working memory, visuoperceptual and visuospatial processing, arithmetic, and general cognition. The full list of tests and the primary cognitive domain tested by each is shown in Table 2, along with abbreviations used in the results section. Descriptive statistics of scores for each test and per patient group are provided in Table S1. We found no significant age‐related effects in any of the cognitive tests, which reassures us that group differences are due to disease.

**TABLE 2:** Cognitive domains tested by our neuropsychological test battery

Cognitive domain	Neuropsychological test	Abbreviation
Vision	A Cancellation time (time)	A Cancel (time)
	A Cancellation time (number missed)	A Cancel (n Miss)
	VOSP Fragmented Letters	Fragmented Letters
	VOSP Dot Counting	Dot Count (n correct)
	Efron Shape Discrimination	Shape Discrimination
	VOSP Object Decision	Object Decision
Memory	Short Recognition Memory Test (Words)	SRMT (W)
	Short Recognition Memory Test (Faces)	SRMT (F)
	Paired Associate Learning	PAL
	Digit Span (Forwards, total)	Digit Span (F)
	Digit Span (Forwards, maximum)	Digit Span (F Max.)
	Digit Span (Backwards, total)	Digit Span (B)
	Digit Span (Backwards, maximum)	Digit Span (B Max.)
General cognition	Mini Mental State Examination	MMSE
Arithmetic	Graded Difficulty Arithmetic (addition)	GDA (add)
	Graded Difficulty Arithmetic (subtraction)	GDA (sub)
	Graded Difficulty Arithmetic (total)	GDA (tot)

For further details on many of these tests, we refer the reader to [31, 32, 33, 34, 35, 36, 37]

### 2.3 Statistical analysis

#### 2.3.1 Event‐based model

The event‐based model^7^ is designed to estimate a data‐driven, probabilistic sequence of biomarker “events” that represents an underlying cumulative process, using a cross‐sectional set of observations. These can be any biomarkers. In the context of neurodegenerative diseases, an event corresponds to a group‐level statistical deviation from normality/health (defined by data from controls) toward abnormality/disease (defined by data from patients), with the full sequence of events representing the cumulative effects of neurodegenerative disease progression. The ordering of events is determined probabilistically, in a data‐driven manner, by pooling biomarker severity (event probability) across individuals. Conceptually, higher prevalence corresponds to an earlier position in the sequence. The event‐based model estimates both the sequence and uncertainty in the sequence. Event probability is a function of the likelihood of an event having occurred $Pr(x_{ij}|E_{i})$ (“post‐event”), or having not occurred $Pr(x_{ij}|\neg E_{i})$ (“pre‐event”), via a two‐component univariate mixture model (see Section 2.3.2). Assuming independent observations and biomarkers, the likelihood of an ordered sequence S is [8]

(1) $$\begin{array}{c} Pr\left(X|S\right)=\prod_{j=1}^{N}\left[\sum_{m=0}^{M}\left\{\prod_{i=1}^{m}Pr\left(x_{ij}|E_{i}\right)\prod_{i=m+1}^{M}Pr\left(x_{ij}|\neg E_{i}\right)\right\}\right] \end{array}$$

where measurements x∈X come from i∈M event markers and j∈N individual samples, such as participants in a disease cohort.

In the absence of prior information, a uniform prior distribution over sequences is used and the characteristic ordering $\hat{S}$ is the sequence that maximizes the likelihood Pr(X|S) in (1). This requires a search among the M! possible sequences, which quickly explodes to preclude an exhaustive search when M exceeds ∼10, necessitating approximation of this maximization. The search is performed using a combination of multiply initialized gradient ascent and Markov chain Monte Carlo sampling.

##### 2.3.1.1 Patient staging

An individual sample $X_{j}$ (vector of measurements across biomarkers i for an individual j) is staged within a given sequence via the data likelihood. For example, by finding the stage m that maximizes the individual likelihood^9^:

(2) $$\underset{m}{\arg\;\max}Pr\left(X_{j}|S,m\right)=\underset{m}{\arg\;\max}\prod_{i=1}^{m}Pr\left(x_{ij}|E_{i}\right)\prod_{i=m+1}^{M}Pr\left(x_{ij}|\neg E_{i}\right).$$

#### 2.3.2 Mixture modeling and our new event‐based model

Disease severity varies among patients, so we fit a two‐component mixture model to determine event probability for each biomarker (cognitive test scores in this work). This probabilistic assignment of individuals into two subgroups — pre‐event and post‐event — allows patients to have combinations of normal and abnormal observations across biomarkers, which in turn allows for the sequence of events to be estimated. Previous event‐based models have incorporated parametric mixture models — most commonly a Gaussian mixture model, which works well for many data types including imaging markers that have been the primary application of the event‐based model to date. However, a Gaussian mixture model can be highly inappropriate for skewed data, such as the cognitive test scores considered here. Indeed, we show in Supplementary Material that Gaussian mixture modeling produces erroneous event sequences, necessitating our alternative, nonparametric mixture model proposed here — see Figures S3, S4, and S5.

We emphasize that events are inherently probabilistic. While we use the language of events having “occurred” or not, this is in an explicitly probabilistic sense. This is one of the event‐based model's key benefits — that explicit biomarker cutpoints are not required. We now discuss how the mixture modeling is used to calculate event probabilities.

##### 2.3.2.1 Parametric, Gaussian mixture modeling

Two‐component Gaussian mixture model fitting involves estimating the mean and variance of each component, and a mixture weight. We initialized the pre‐/post‐event components using the diagnostic labels control/diseased. We initialized equal mixture weights of 0.5 and would terminate the fit if either weight exceeded the range [0.1,0.9] to ensure that components do not vanish when the data from diagnostic groups overlap considerably. Parameters were optimized to minimize the negative log‐likelihood of the data given the mixture model, using the sequential least‐squares programming (SLSQP) algorithm. These choices of constraints, initial parameters, and the SLSQP algorithm are similar to the classic event‐based model.^8^, ^9^

##### 2.3.2.2 Non‐parametric, kernel density estimation mixture modeling

Kernel density estimation (KDE) is a non‐parametric method for estimating a probability density. The KDE estimate $\hat{f}\left(x\right)$ of a function f(x) with an independent and identically distributed sample $\left\{x_{j}\right\}$ drawn from a distribution with an unknown density is given by

(3) $$\begin{array}{c} \hat{f}\left(x\right)=\frac{1}{Nh}\sum_{j=1}^{N}K\left(\frac{x-x_{j}}{h}\right), \end{array}$$

where K is a non‐negative zero‐mean kernel function that integrates to unity and h is a positive smoothing factor called a bandwidth. With an appropriate choice of K, KDE naturally extends to multivariate density estimation. In this work we estimate mixture model components using the KDE implementation in scikit‐learn,^38^ with a default (Gaussian) kernel and default parameters for all values except the bandwidth (see below). The choice of kernel function K is not dependent upon the datatype of the biomarker being modeled. For example, there is no restriction against using a smooth kernel function when modeling discrete‐valued cognitive test scores.

The KDE approach enables data‐driven estimation of probability densities that do not follow a parametric distribution, which is particularly useful for cognitive test scores that are susceptible to ceiling effects and floor effects. Our novel mixture modeling algorithm incorporates non‐parametric mixture modeling using KDE components. As for our Gaussian mixture modeling, we initialized the mixture model components using the labeled data (HC, diseased), and start with equal mixture weights that are restricted to values within the range [0.1,0.9] to ensure that components do not vanish. We choose to fix the biomarker bandwidth h using Scott's normal reference rule^39^ applied jointly on both groups. A variable bandwidth would be sensible for KDE of highly skewed data with sparsely populated tails. Our full algorithm is given in the Supplementary Material and code is available at https://github.com/noxtoby/kde_ebm_open.

#### 2.3.3 Cross‐validation

We performed cross‐validation of our event‐based models by re‐estimating each full model (event distributions and maximum‐likelihood sequence) on 100 bootstrap samples (sampling with replacement). The resulting bootstrapped model tends to overestimate positional variance in the event sequence.

## 3 RESULTS

Here we present results from our experiments on real data in two dementia syndromes using our new event‐based model incorporating a KDE‐component mixture model. In Figures S6–S8 we present a comparison with the existing state of the art — the classic event‐based model^9^ incorporating a Gaussian mixture model. Detailed performance evaluation results from our simulation experiments are in Figures S3–S5, where we show that our new method outperforms the state of the art method in situations where the underlying biomarker distributions are similar to those observed from cognitive tests.

### 3.1 Sequence of cognitive decline in PCA

**FIGURE 1:**
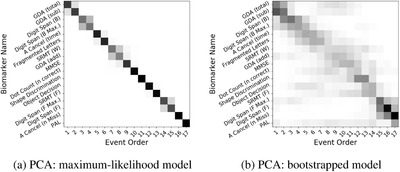
Data‐driven sequence of cognitive decline in posterior cortical atrophy (PCA): (a) Maximum‐likelihood model; (b) Bootstrapped model. The bootstrapped model overestimates positional variance in the sequence (see Materials and Methods, Section 2). Grayscale intensity represents the proportion (0 in white, 1 in black) of the posterior Markov chain Monte Carlo samples in which events (y‐axis) appear in a particular position (x‐axis) in the sequence

Figure 1 is a visualization of the probabilistic sequence of detectable cognitive decline due to PCA that was estimated by our data‐driven method, using the set of neuropsychological tests in our battery. The posterior positional variance shows the model's confidence (left‐to‐right) in the ordering (top‐to‐bottom): narrow, dark sections of low positional variance show high confidence in the ordering. Figure 1A (left) shows the maximum‐likelihood model and Figure 1B (right) shows a more conservative estimate of the variability in the sequence using bootstrapping.

In agreement with clinical knowledge, our results suggest that measures of visual processing become abnormal earlier in PCA patients (A Cancellation (time) and VOSP Fragmented Letters), while the model estimates relatively late deficits in episodic memory (Short Recognition Memory Test [SRMT] and Paried‐Associate Learning [PAL]), although in the case of PAL this may be influenced by missing data (see Supplementary Material). The location of PAL does not affect the ordering of other events. Deteriorating performance on an arithmetic test (GDA) was estimated to be the earliest event, although the bimodal nature of the positional density under bootstrapping (Figure 1B, top rows) may reflect heterogeneity within PCA. Tasks having higher demands on working memory (Digit Span backward) appeared earlier than simpler short‐term memory tasks (Digit Span forward), although the latter is less certain as indicated by the weakly bimodal positional density under bootstrapping.

Our model produces results more consistent with intuition and expectations than the classic event‐based model (Figures S3–S9). Specifically, in our model the highly correlated subscores (total items correct and maximum span) of both the forward (F, F max.) and backward (B, B max.) Digit Span tests appear in successive positions in the event sequence, as expected.^40^ The same cannot be said of the classic event‐based model incorporating a Gaussian mixture model (Figure S6) where these subscores are almost maximally separated in the sequence. In the case of GDA subscores, the situation may appear to be reversed (our model separates correlated subscores, while the classic event‐based model does not) until one considers the aforementioned bimodal nature of the bootstrapped density for GDA in Figure 1B. This warrants further investigation.

### 3.2 Sequence of cognitive decline in tAD

**FIGURE 2:**
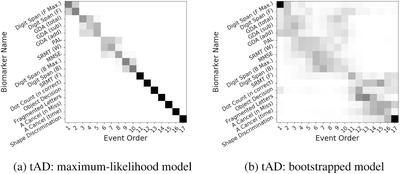
Data‐driven sequence of cognitive decline in typical Alzheimer's disease (tAD): (A) Maximum‐likelihood model; (B) Bootstrapped model. See Figure 1 for further explanation of positional variance diagrams

Figure 2 is a visualization of our method's data‐driven sequence of detectable cognitive decline due to tAD, using the same set of neuropsychological tests as for PCA in Figure 1.

In agreement with clinical knowledge, our results suggest that tAD patients show early deterioration in episodic memory (PAL and SRMT) with visual processing deficits clearly confined to the end of the sequence, even in the more‐variable bootstrapped model of Figure 2B. This is largely a reversal of the sequence estimated for PCA, with the exception of GDA which was among the earliest events in both dementia syndromes (see Discussion in Section 4). Digit Span was also involved very early, although the bootstrapped model contains bimodal positional density either side of episodic memory (PAL and SMRT), which may reflect heterogeneity within tAD.

### 3.3 Patient staging and longitudinal self‐consistency of models

We use patient staging to assess model self‐consistency. A valid model consistent with the progressive nature of dementia will produce non‐decreasing disease stages for individuals from baseline to follow‐up(s), within model uncertainty. Our disease progression models are trained on baseline data, with longitudinal self‐consistency assessed on follow‐up data (a separate test set, albeit from the same individuals). Described mathematically in Section 2, within each dementia syndrome we assigned an event‐based model stage to each individual using Equation 2, which is akin to aligning an individual's cognitive profile (test scores) with the maximum‐likelihood model. We emphasize that these are self‐consistency checks within a model which are not useful for direct model comparison purposes.

**FIGURE 3:**
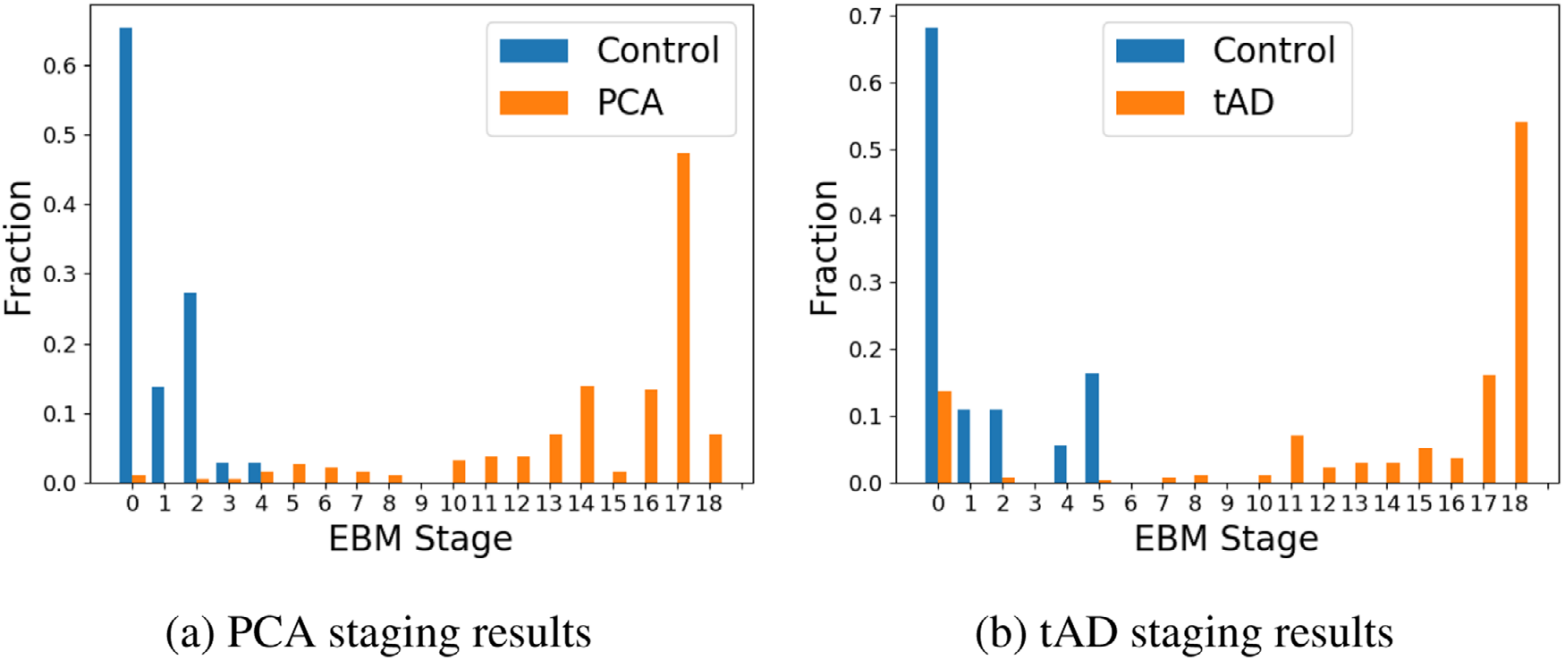
Histogram of model stages assigned to healthy controls and patients with posterior cortical atrophy (PCA) and typical Alzheimer's disease (tAD)

Figure 3 summarizes the distribution of event‐based model stages in PCA (left) and tAD (right) as histograms. As expected, HC generally perform better across all tests and are assigned to early stages. Commensurately, patients generally exhibit poorer performance and are assigned later stages — mostly very late stages. The only notable exception is a small fraction of tAD patients who have been assigned a very early event‐based model stage in Figure 3B. These four patients have mild symptoms (MMSE score ≥24, mean 25.7) and all performed considerably better than other patients on Digit Span Forwards and GDA (the earliest events), which dominated the mild abnormality in later events including MMSE.

Quantitatively, our models show high longitudinal self‐consistency. We calculate the proportion of pairs of visits within individuals having non‐decreasing model stage, within model uncertainty (positional variance). In PCA we found 244 of 262 available follow‐up combinations to be consistent (93%). In tAD we found 34 out of 39 (89%). These self‐consistency results are not influenced by baseline stage (early vs late) because the number of visits per patient is comparable across baseline stages.

## 4 DISCUSSION

We have revealed fine‐grained representations of deterioration across cognitive domains in two dementia syndromes: PCA and tAD. This was enabled by a novel event‐based model for estimating data‐driven sequences of cognitive decline in neurodegenerative diseases, as well as uncertainty in the sequences.

We estimated sequences of cognitive decline that are broadly consistent with clinical criteria for, and reports of progression in, PCA and tAD. This supports the use of our method in biological applications where high‐precision staging is required, such as for screening in clinical trials. Decline in the five‐principle visual tests was estimated to occur earlier (A cancellation time, Fragmented Letters, Dot Counting, Shape Discrimination), or at a comparable stage (Object Decision), in PCA relative to tAD (mean 5.6±4.2 positions earlier, among 17 possible positions). Likewise, decline in the three‐principle episodic memory tasks (short Recognition Memory Test for Words and Faces, and the Paired Associate Learning test) was estimated to occur somewhat earlier in tAD than PCA (mean 4.3±5.9 positions earlier). Arithmetic performance was estimated to decline early in both patient groups, which might relate to this graded task being designed both to obtain a normal (Gaussian) distribution of scores and also to avoid floor and ceiling effects. Furthermore, the timed nature of the Graded Difficulty Arithmetic (GDA) test makes it sensitive to general decline in attention and processing speed (affected in both dementia syndromes), in addition to acalculia.

We validated our models by assessing longitudinal self‐consistency of patient staging on a separate test set: the follow‐up data in each cohort. In PCA, we observed 93% longitudinal consistency (non‐decreasing patient stage at follow‐up), and in tAD we observed 89%.

We further assessed the robustness of our results by comparing the maximum‐likelihood models with their bootstrapped counterparts, the latter of which tends to overestimate uncertainty. The estimated sequences are robust, with the 2D positional density maps remaining consistently close to the diagonal.

A number of previous studies in PCA found broadly supportive results. Our finding that visual and visuospatial deficits (eg, A Cancellation time/Object Decision) precede those in memory (MMSE) was also found in refs. [40, 41, 42]. Our finding of early mathematical difficulty (GDA) agrees with ref. [42].

In Supplementary Material we demonstrated the superiority of our new event‐based model over the classic event‐based model which incorporates Gaussian mixture modeling (in simulation and in application to real data), especially for highly non‐Gaussian data such as found in standard neuropsychological tests where ceiling effects and floor effects are common. When the input data are close to Gaussian, both models perform well and the classic event‐based model is probably the better choice for model parsimony (see Supplementary Material). We note that our new model is capable of working “out of the box” on both Gaussian and non‐Gaussian data, ameliorating the need for preprocessing steps traditionally used on non‐Gaussian data, such as log transformation. However, it would be straightforward to add an extra model‐selection step to choose the most appropriate mixture‐model for each biomarker if desired.

We highlight some strengths and limitations of our study. First, a particular strength of our methodology, by design, is its flexibility to handle non‐Gaussian data such as clinical test scores. This was verified by our extensive performance evaluation experiments in the Supplementary Material. Secondly, our neuropsychological battery intentionally tests cognitive domains specific to PCA and tAD, which is a strength in terms of both specificity and for comparing two dementia syndromes, but is a minor limitation in terms of discovery — that is, our data cannot discover whether an untested domain/function such as language or motor function is useful in these syndromes. As noted in many of the original publications on the method,^8^, ^9^ one limitation of the event‐based model (in general, not our particular model) is the assumption of events being independent, which is not the case for cognitive tests which will often depend upon multiple domains to varying extent. Interpretation of results must keep this in mind.

Considering the rarity of PCA, our sample sizes are good, although modest. The event‐based model is relatively robust to small sample sizes due to (1) its simplicity (the minimum viable sample size is simply the number of features); and (2) the model being designed to highlight uncertainty (through the positional variance diagram). In our experiments the minimum viable sample size was exceeded, often substantially (17 features, 20–94 observations per feature), resulting in the precision we observed. The effect of a small sample size on an event‐based model is to increase uncertainty in the ordering of events, which would manifest as increased positional variance such as in the bootstrapped models in Figures 1B and 2B. A key advantage of the event‐based model is that it explicitly highlights this uncertainty to reveal parts of the progression where the data may be insufficient to inform on the precise sequence.

Our results promote further clinical investigation of cognitive assessment in dementia, such as the early estimated abnormality of the A Cancellation task in PCA. This suggests that measures intended to assess executive function/attention but which feature a prominent visual search component (eg, Trailmaking tasks, Digit Symbol) are susceptible to visual processing deficits.

Our results motivate us to consider avenues for future work in both applications and methodological directions: characterizing cognitive decline in other neurodegenerative diseases (eg, Parkinson's dementia, Huntington's disease, familial Alzheimer's disease), including investigating the possibility of cognitive subtypes; application of such results in clinical trials; and working toward turning KDE mixture modeling into a more general statistical methodology. The first avenue will improve disease understanding, for which we foresee wider application of our new method on larger datasets involving more individuals and/or a more extensive battery of neuropsychological tests. Although the method has already been used in a few studies,^26^, ^43^, ^44^, ^45^ future work includes application to other multimodal datasets (eg, cognitive and imaging data) to reveal insights into the progression of neurological diseases. This will include embedding our method within the classic event‐based model,^8^, ^9^ within the recently proposed discriminative event‐based model,^46^, ^47^ and within data‐driven disease subtyping algorithms such as Subtype and Stage Inference (SuStaIn).^15^ The second avenue for future work turns this improved disease understanding into quantitative tools for clinical trials. This includes enrichment through precise stratification and subject selection, and using model stage as a data‐driven composite cognitive end‐point. This is ongoing work. The third avenue involves methodological advances, for which we envisage starting with performance characterization on more complex clustering tasks — our formalism is quite general but our experiments involved only two components/clusters in the mixture model. Such investigations might also include application to feature normalization for supervised learning. Additionally, embedding KDE mixture modeling within a Bayesian framework would broaden its utility and enable the use of weakly informative priors on hyperparameters for constraining the clustering results. Such constraints can be important for ensuring realistic results in biological applications.^48^

In summary, our results verify clinical opinion on disease progression while revealing new insight into the fine‐grained sequence of clinical decline in memory‐led tAD and vision‐led PCA. This fine‐grained understanding promises clinical utility for informing earlier differential diagnosis, and prognosis through data‐driven disease staging — both are relevant for clinical trials and patient management.

## FUNDING

The funding agencies had no involvement in the research itself, including: study design; data collection, analysis, and interpretation; writing of this report; nor the decision to submit this article for publication.

The authors declare that the research was conducted in the absence of any commercial or financial relationships that could be construed as a potential conflict of interest.

## Supporting information

Supporting Information
