# Supplementary material for: Sequences of cognitive decline in typical Alzheimer's disease and posterior cortical atrophy estimated using a novel event‐based model of disease progression
Source: Alzheimers Dement. 2020 Jun 2;16(7):965–73. doi: 10.1002/alz.12083 (PMC8432168; doi:10.1002/alz.12083)
Supplement: Supplementary file 1 — Supporting Information [file ALZ-16-965-s001.pdf]

Supplementary Material: Sequences of cognitive decline in typical Alzheimer's disease and posterior cortical atrophy estimated using a novel event-based model of disease progression

Nicholas C. Firth[a,b], Silvia Primativo[c], Emilie Brotherhood[b], Alexandra L. Young[a], Keir X.X. Yong[b], Sebastian J. Crutch[b], Daniel C. Alexander[a,d], Neil P. Oxtoby[a,*]

[a] *Centre for Medical Image Computing, Department of Computer Science, UCL, London, WC1E 6BT, U.K.*
[b] *Dementia Research Centre, UCL Queen Square Institute of Neurology, UCL, London, WC1N 3BG, U.K.*
[c] *Department of Human Science, LUMSA University, Rome, Italy*
[d] *Clinical Imaging Research Centre, National University of Singapore, Singapore*

Abstract

Supplementary material.

Abbreviations: PCA: Posterior Cortical Atrophy; tAD: typical Alzheimer's disease; HC: Healthy Controls; VOSP: Visual Object and Space Perception (battery); MMSE: Mini Mental State Examination; SRMT: Short Recognition Memory Test; PAL: Paried-Associate Learning; GDA: Graded Difficulty Arithmetic; Obj.: Object; Fragm.: Fragmented; SD: standard deviation; KDE: kernel density estimate/estimation; GMM: Gaussian mixture model/modeling; KDEMM: KDE mixture model/modeling; ML: maximum-likelihood; MCMC: Markov Chain Monte Carlo; EBM: event-based model

*Corresponding author.

URL: neiloxtoby.com (Neil P. Oxtoby)

This document contains supplementary material including: descriptive summary of scores for each test in our neuropsychological battery (Supplementary Section 1); mathematical details of our nonparametric mixture model (Supplementary Section 2); and detailed performance evaluation experiments of our new event-based model using both synthetic and real data (Supplementary Section 3).

1. Neuropsychological test battery

Supplementary Table 1 contains a descriptive summary of performance on each test in our neuropsychological battery, for each patient group. Sample sizes are also shown (not all tests were able to be performed by all study participants). Missing data is handled in the event-based model as described in [1], which imputes missing data with values that do not bias the model estimation. Briefly, this is performed after mixture modelling, using values that correspond to 50% abnormality (equally normal and abnormal). We expand on missing data in Supplementary Section 3.5.

	Max score	**PCA** ($n = 94$)		**tAD** ($n = 61$)		**HC** ($n = 23$)	
		n	mean (SD)	n	mean (SD)	n	mean (SD)
MMSE	30	89	20.88 (5.17)	56	19.58 (5.08)	22	29.02 (0.98)
SRMT (faces)	25	67	18.75 (4.12)	39	18.76 (4.69)	23	24.61 (0.94)
SRMT (words)	25	93	19.91 (3.79)	40	16.21 (3.38)	23	24.52 (0.85)
PAL	24	21	10.74 (7.57)	20	3.26 (6.01)	23	20.52 (3.48)
Digit Span (fwd)	12	72	6.0 (2.96)	59	6.23 (2.17)	23	8.78 (1.73)
Digit Span (bwd)	12	71	3.39 (1.86)	60	3.98 (2.37)	23	7.96 (1.49)
GDA (addition)	12	62	1.31 (2.23)	54	2.47 (2.7)	23	7.17 (3.14)
GDA (subtraction)	12	62	0.91 (1.95)	54	2.06 (2.92)	23	7.0 (3.85)
Basic Vision							
Shape discrimination	20	89	14.78 (3.38)	58	17.73 (3.05)	22	19.41 (0.8)
Space Perception							
Dot counting (VOSP)	10	91	4.92 (3.36)	57	8.26 (2.64)	23	9.91 (0.29)
A cancel. (time)	90	88	79.23 (31.78)	55	44.19 (21.4)	23	19.87 (4.66)
A cancel. (n missed)	21	90	4.68 (4.85)	58	0.93 (1.56)	23	0.16 (0.37)
Object Perception							
Obj. decision (VOSP)	20	94	10.37 (4.58)	59	16.34 (2.38)	23	18.06 (1.65)
Fragm. letters (VOSP)	20	82	5.15 (5.49)	58	14.36 (6.07)	22	19.5 (0.67)

Supplementary Table 1: Details of the neuropyschological battery, including sample sizes per test.

2. Nonparametric mixture modeling with KDE components

Let $x_j \in \mathbb{R}^N$ be a set of N observations, and $S_1^{(1)}, S_2^{(1)}, \ldots, S_\nu^{(1)}$ be ν subsets of the data, known at iteration step $t = 1$. Let h be the bandwidth of the full set, which we estimate using Scott's normal reference rule [2]. We initialize equal mixture weights

for all subsets: $w_1^{(1)} = w_2^{(1)} = \cdots = w_\nu^{(1)} = 1/\nu$. Similar to the k-means clustering algorithm, our algorithm iterates over alternating assignment and update steps to optimize parameters, as shown in Supplementary Figure 1.

Update Step: At algorithm iteration t, for each subset $S_\nu^{(t)}$ a KDE mixture component $\hat{f}_\nu(x_j)$ is fit using

$$\hat{f}_\nu(x_j) = w_\nu^{(t)} \frac{1}{Nh} \sum_{x_\nu \in S_\nu} K\left(\frac{x_\nu - x_j}{h}\right). \tag{1}$$

Assignment Step: Each observation is then assigned to a new subset $S_\nu^{(t+1)}$, to which it has the maximum likelihood of belonging from $\hat{f}_\nu(x_j)$. Mixture weights are then updated to be the proportion of observations in each subset, i.e., $w_\nu^{(t+1)} = |S_\nu^{(t+1)}| / \sum_{c=1}^{v} |S_c^{(t+1)}|$.

The update and assignment steps are iterated until subset assignment is no longer updated, i.e., $S_\nu^{(t)} = S_\nu^{(t+1)}$, $\forall\, \nu$. As in our Gaussian mixture modeling, we restrict the mixture weights to values $0.1 < w_\nu < 0.9$, $\forall\, \nu$ to ensure that subsets do not vanish.

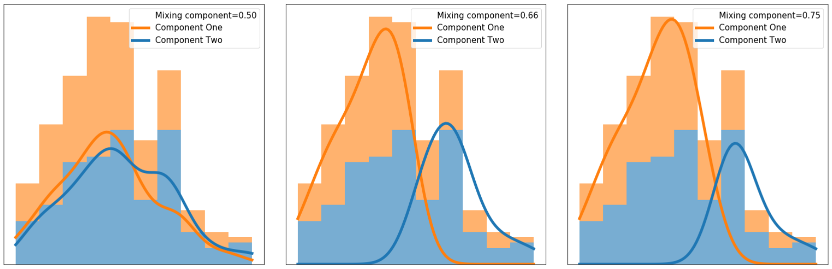

(a) Update step for first iteration

(b) Iteration 2

(c) Iteration 7 (converged)

Supplementary Figure 1: Example fitting process for a non-parametric mixture model with KDE components.

3. Performance evaluation of our new event-based model

3.1. Simulation experiments

We evaluated the performance of our method using $D = 25$ synthetic datasets each consisting of $N = 200$ individuals and $M = 3$ event markers. Two subpopulations of individuals were created, labelled HC ($n = 100$) and AD ($n = 100$) to correspond to healthy controls and individuals with a disease, respectively. The event marker values are synthesized from subpopulation densities of varying overlap/separation and varying Gaussianity (described below). The goal of the mixture modeling is to estimate, for each marker, the two distributions corresponding to an event having occurred or not occurred (Supplementary Figure 2). For each dataset a random event sequence was generated, and the progression of biomarkers from normal to abnormal/disease

followed this event sequence. For the HC subpopulation 55% of individuals were assigned to stage zero (no marker events have occurred), 25% at stage one (only the first event has occurred), 15% at stage two (the first two events have occurred) and 5% at stage three (all events have occurred). For the AD subpopulation this trend was reversed with 55% at stage three, 25% at stage two, 15% at stage one and 5% at stage zero.

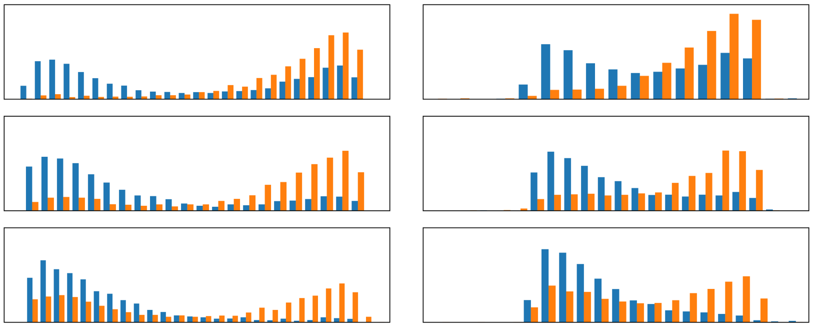

(a) High skew, high separation (b) High skew, low separation

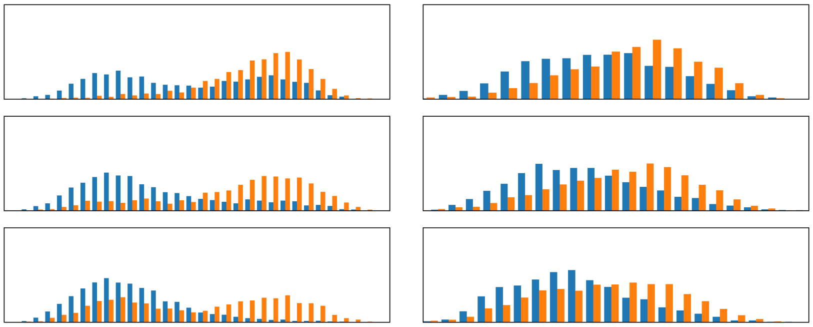

(c) Low skew, high separation (d) Low skew, low separation

Supplementary Figure 2: Histograms of exemplar synthetic data using Gamma-distributions. Skew and separation decreases from upper-to-lower and left-to-right: (a) $\kappa = 1.5$, $f = 2$; (b) $\kappa = 1.5$, $f = 1$; (c) $\kappa = 10$, $f = 1.5$; (d) $\kappa = 10$, $f = 0.5$. Note: κ is the Gamma distribution shape parameter; f is the separation factor (see text).

To test our method against Gaussian mixture modeling we simulated event marker data having varying levels of Gaussianity (high to low) and group separation (high to low), as observed in disease cohorts. For this purpose, we used Gamma distributions to synthesize two-component marker data of varying shape profiles κ and separation/overlap f. Using the `scipy` library [3], two sets of Gamma-distributed random numbers were generated with unity scale. To mimic disease progression, both were mean-centred and one was reflected (multiplied by negative unity) then shifted by adding f. The separation factor $f = 2\phi\sigma_\kappa$ is a controlled multiple (2ϕ) of the standard deviation σ_κ of the Gamma distribution.

We used three quantitative measures to compare the mixture modeling approaches:

1. likelihood of the event marker data given the mixture model;
2. correlation of the estimated event-based model sequence with the ground truth; and
3. correlation of the data-driven staging of individual samples with the ground truth.

For 1 we used the likelihood-ratio test to compare goodness of fit for each mixture model, with the Gaussian mixture model representing the null hypothesis. For 2 we used Kendall τ rank correlation, a non-parametric test of how similar two rankings/sequences are: $\tau = -1$ indicates that one sequence is the mirror opposite of the other and $\tau = 1$ that the two sequences are identical. For 3 we used Spearman's rank correlation to compare the accuracy of the maximum-likelihood stage (Section 2.3.1 (main manuscript)) with the ground truth stage used to generate the data.

3.2. Simulation experiment results: performance evaluation of old and new methods

We synthesized 5400 event markers each containing two components representing patients and controls: three markers in each of 25 datasets, for 72 differently-shaped Gamma distributions (Supplementary Section 3.1) — parameters ϕ ($n = 8$) and k ($n = 9$). For each of the 5400 ($3 \times 25 \times 8 \times 9$) event markers, the data likelihood conditioned on each mixture modeling approach was calculated. Supplementary Figure 3a shows that the Gaussian mixture model has a higher likelihood than the KDE mixture model for most parameter combinations, particularly for high skew and high separation (lower left of the heat map). This may be an artefact of the small standard deviation of the data (which amplifies Gaussian likelihoods) or of the nature of the GMM fitting procedure (which is designed to maximize this likelihood). Regardless, Supplementary Figure 3b suggests that the likelihood differences are largely insignificant. The heat map in Supplementary Figure 3b shows p values for a likelihood ratio test where the GMM is the null hypothesis (numerator) and our new KDEMM is the alternative. Red suggests that the null hypothesis can be rejected at the $\alpha = 0.05$ level, blue that the null should probably not be rejected.

As the intended use for these mixture models is to predict probability of an event having occurred in an event-based model, we focus our performance comparison of the GMM and KDEMM within the context of an event-based model. Synthetic datasets were generated with random event sequences, and for each model the characteristic event sequence was generated by enumerating all possible sequences. Supplementary Figure 4 shows the difference between the GMM and KDEMM event-based models in Kendall τ correlation coefficient with ground truth. It can be seen that the event-based model using the KDEMM has a significantly higher correlation ($\tau = 0.89$) with the ground truth order of events compared to event-based model using GMM ($\tau = 0.50, p < 1\text{e}{-90}$). This trend is seen across the majority of parameters used to create the synthetic datasets. However the GMM performs equally well when there is a sufficiently large separation between components and large enough shape parameter (thus reducing the skewness, i.e. more similar to Gaussian). The GMM performs better in only three parameter combinations, and in only one of these, $\phi = 10$ and $k = 10$, does the corresponding event-based model perform significantly better.

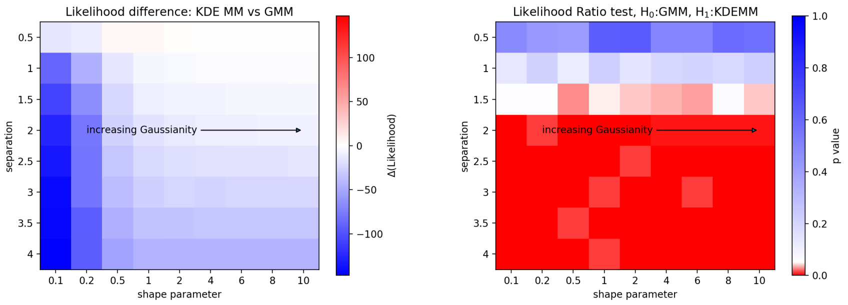

(a) Likelihood difference

(b) Likelihood ratio test results

Supplementary Figure 3: Heat map comparing data likelihoods across parameter space given two mixture models. Red regions favour the KDEMM and blue regions favour the GMM. (a) shows the difference in likelihoods. (b) shows the results (p values) of a likelihood ratio test to determine statistical significance of the differences in (a).

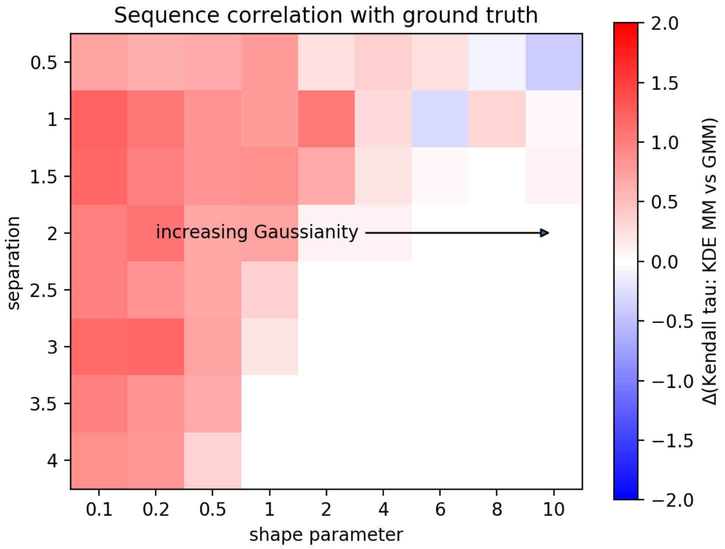

Supplementary Figure 4: Heat map comparing model sequence correlation (Kendall τ) with simulated ground truth across parameter space. Red regions favour the KDEMM-based model (positive $\Delta\tau$) and blue regions favour the GMM-based model (negative $\Delta\tau$).

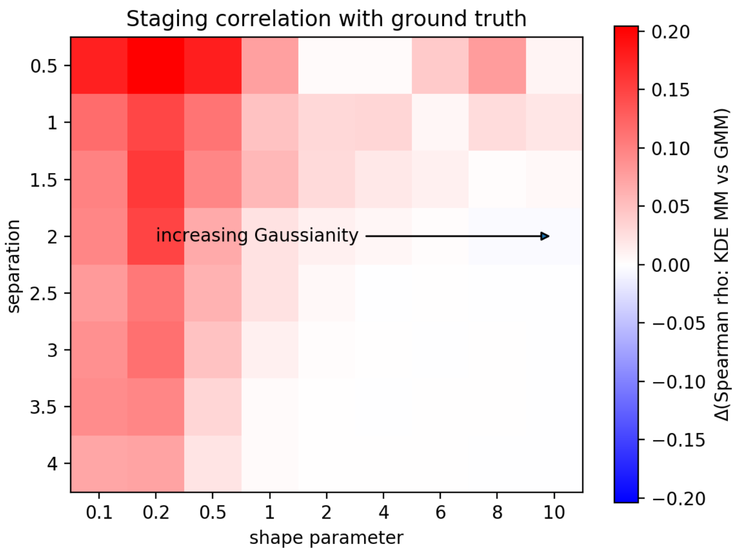

Supplementary Figure 5: Heat map comparing model staging correlation (Spearman ρ) with simulated ground truth across parameter space. Red regions favour the KDEMM-based model (positive $\Delta\rho$) and blue regions favour the GMM-based model (negative $\Delta\rho$).

As well as using the event-based model for generating an event sequence, it has also been used to give a disease stage for study participants. Supplementary Figure 5 shows a comparison between modelled stages from GMM- and KDEMM-event-based models built using the ground truth stage. It can be seen that the event-based model stages using the KDEMM model, correlated with the ground truth stages ($\rho = 0.88$) significantly better than the event-based model stages using the GMM model ($\rho = 0.83, 1\text{e}{-18}$). Similarly to the event sequence the GMM staging performs equally well when the data was generated with a sufficiently large separation factor and shape parameter. This likely is due to the staging reliance on an accurate event order as well as well-fit mixture models.

3.3. Experiments on real data: comparison of old and new methods

Using both the GMM and KDEMM within the event-based model for cognitive data, we see different distinct event orderings. In the PCA Maximum Likelihood (ML) sequence fit using GMM models the Digit Span (F) and Digit Span (F Max.) are separated by 13 positions in the event sequence (Supplementary Figure 6a); this is contrary to previous results [4] and intuition, which would suggest that subscores of the same cognitive test would occur at very similar positions in the event sequence. The ordering of cognitive tasks also does not fit with clinical expectations, with PCA patients showing earlier memory change (mean 33.2 ± 2.6 positions earlier) compared to typical Alzheimer's disease and no difference in visual change (mean 0.4 ± 5.9 positions ear-

lier) compared to typical Alzheimer's disease patients. By comparison, ML sequences fit for both typical Alzheimer's disease and PCA using the KDEMM model yielded successive positions for the highly related Digit Span (F) and Digit Span (F Max) scores. As well as being clustered next to each other in the sequences, their positional variance overlaps in both the maximum-likelihood model (main manuscript: Figure 1a, Figure 2a) and conservative model from bootstrapping (main manuscript: Figure 1b, Figure 2b). For both PCA and typical Alzheimer's disease, the order of cognitive events estimated using the KDEMM approach (main manuscript: Figure 1, Figure 2) aligns much better with clinical definitions than the order estimated using the GMM approach (Supplementary Figures 6 and 7). In both the GMM and KDEMM event-based models there is notable uncertainty in the bootstrapped sequences, however in the GMM this appears to be distributed away from the ML sequence, as observed by the spread away from the diagonal (Supplementary Figure 6b and Supplementary Figure 7b), whereas in the KDEMM event-based model the uncertainty is more focused on the diagonal (main manuscript: Figure 1b, Figure 2b), suggesting that the sequence is more robust (under bootstrap cross-validation). We conclude that the broad uncertainty in the conservative models in the GMM approach is likely due to non-Gaussianity of the component distributions.

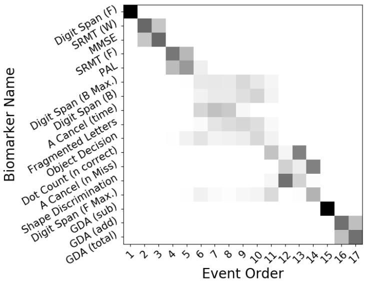

(a) PCA: maximum-likelihood model

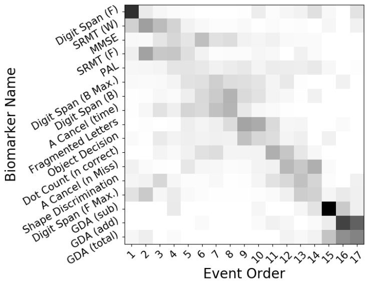

(b) PCA: conservative model

Supplementary Figure 6: Data-driven sequence of cognitive decline in PCA using the classic event-based model, incorporating a Gaussian mixture model: (a) Maximum-likelihood model; (b) Conservative model. The conservative model over-estimates positional variance in the sequence using bootstrapping (see Materials and Methods section). Grayscale intensity represents the proportion (0 in white, 1 in black) of the posterior MCMC samples in which events (y-axis) appear in a particular position (x-axis) in the sequence. Compare this with Figure 1 (main manuscript), estimated using our new event-based model incorporating KDE mixture modeling.

3.4. Experiments on real data: self-consistency of models through patient staging

As described in the Methods section of the manuscript body, each participant is assigned a numerical disease stage calculated from the likelihood of their data given the model. Supplementary Figure 8 shows the distribution of individual model stages assigned by the classic event-based model (left) and our new event-based model (right) for the baseline data used to train the models in typical Alzheimer's disease (lower) and PCA (upper). Model stage is consistent with clinical diagnosis, as evidenced by controls being assigned lower/earlier stages, and patients assigned higher/later stages.

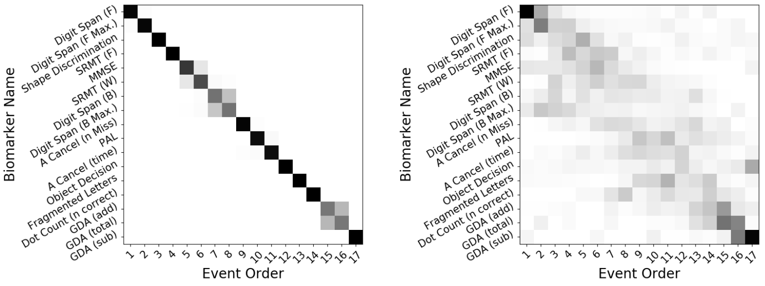

(a) tAD: maximum-likelihood model (b) tAD: conservative model

Supplementary Figure 7: Data-driven sequence of cognitive decline in typical Alzheimer's disease using the classic event-based model, incorporating a Gaussian mixture model: (a) Maximum-likelihood model; (b) Conservative model. Other details as in Supplementary Figure 6.

Notable exceptions are a small number of controls assigned to later stages in the classic event-based model (Supplementary Figure 8a, Supplementary Figure 8c) and a small fraction of mildly-symptomatic typical Alzheimer's disease participants ($n = 4$; MMSE ≥ 24) who were assigned early stages in the KDEMM event-based model (Supplementary Figure 8d). These four mildy-symptomatic patients all performed considerably better than other patients on Digit Span Forwards and GDA (the earliest events), which dominated the mild abnormality in later events including MMSE. To analyze the self-consistency of the fitted models for PCA and typical Alzheimer's disease we staged each of the subsequent followup visits for all patients having longitudinal data ($n = 70$ of 94 PCA; $n = 29$ of 61 typical Alzheimer's disease). The median followup interval was 1.52 years in the PCA cohort and 1.28 years in the tAD cohort, with the distribution of time intervals shown in Supplementary Figure 9. These data were not used to fit the event-based model, so provide a suitable test set. We define staging self-consistency of a model as the percentage of followup visits that are staged no earlier than previous visits (all combinations), while allowing for model uncertainty.

Staging self-consistency of the classic event-based model was 93% (243 of 262) for PCA and 95% (37 of 39) for typical Alzheimer's disease. Staging self-consistency of our new event-based model was 93% (244 of 262) for PCA and 87% (34 of 39) for typical Alzheimer's disease. We emphasize that these are self-consistency checks within a model which are not useful for direct model comparison purposes.

3.5. Missing data

The level of missing data per cognitive test is shown in Supplementary Section 1. Most notably, there is considerable missing data for the Paired Associate Learning (PAL) test: 21 of 94 in our PCA cohort and 20 of 61 in our typical Alzheimer's disease cohort. To confirm that our missing data imputation strategy from [1] did not bias the estimated models, we repeated the real data experiments of Supplementary Section 3.3 with PAL omitted. The sequences were identical (results not shown). Additionally, patient staging results were comparable and longitudinal self-consistency of the models was not affected.

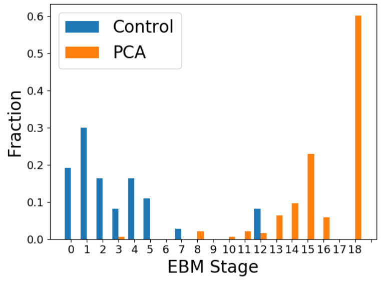

(a) PCA, classic EBM (GMM)

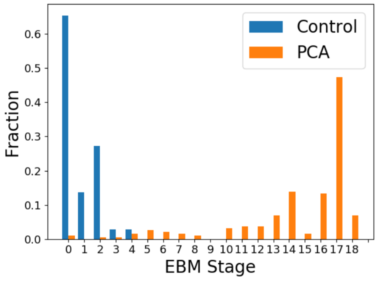

(b) PCA, new EBM (KDEMM)

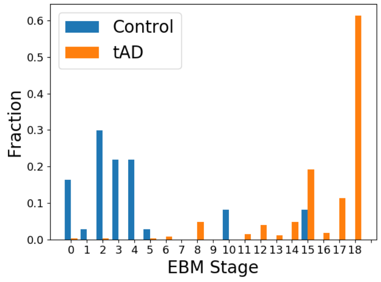

(c) tAD, classic EBM (GMM)

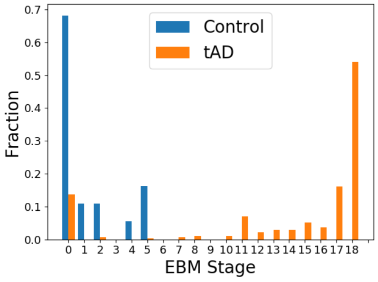

(d) tAD, new EBM (KDEMM)

Supplementary Figure 8: Method comparison of patient staging on real data. Histograms of the maximum likelihood event-based model stages assigned to healthy controls and patients with PCA and typical Alzheimer's disease.

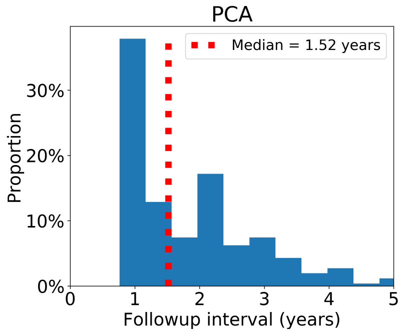

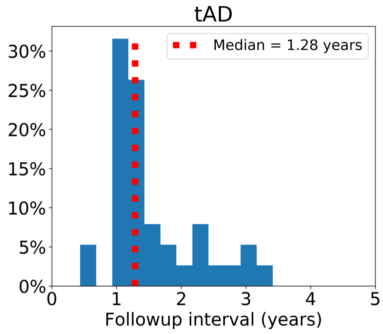

Supplementary Figure 9: Model self-consistency. Distribution of time between stages/visits for each condition: posterior cortical atrophy (PCA, left) and typical Alzheimer's disease (tAD, right).

We also investigated whether the position of PAL within the models might be affected by missing data. A possible cause of this would be if the missing data was due to disease severity, i.e., patients being unable to complete the PAL test. In this hypothetical scenario, PAL would appear later in the estimated model sequence than it should. To test this hypothesis we statistically compared PAL "responders" with "non-

responders" using MMSE as a proxy for disease severity (for which we have the most data in each cohort). Specifically, we performed a Mann-Whitney U test. In our tAD cohort we found $u = 331$ ($p = 0.11$), which suggests that the PAL non-responders (mean MMSE of 19) were not more impaired than the PAL responders (mean MMSE of 21). In our PCA cohort we found $u = 553$ ($p = 0.02$), which suggests that PAL non-responders (mean MMSE of 21) indeed were more impaired than PAL responders (mean MMSE of 24). This suggests that missing data might be a factor in the position of PAL (last) in our event-based model of cognitive decline in PCA but not in typical Alzheimer's disease. This is supported by our bootstrapped model in Figure 1b of the main manuscript, which shows uncertainty in the position of PAL. We surmise that the PAL test may be too difficult for advanced PCA. Further investigation is warranted.

3.6. Discussion

Our synthetic experiments within the context of data-driven disease progression modeling in the event-based model framework [5–7] showed that our method provides better accuracy than the vanilla approach of Gaussian mixture modeling. Broadly speaking, improvement in accuracy was more pronounced when the component distributions were more skewed (less Gaussian) and more overlapping, but KDE mixture modeling performed well even when the component distributions were approximately Gaussian. We conclude that KDE mixture modeling is appropriate for general application in this arena and may potentially replace the Gaussian mixture model as a go-to statistical workhorse of clustering.

Our experiments on real data from neuropsychological test scores in two dementias provide strong support for the use of our method in biological applications, particularly in neurology. Our method produced models of cognitive decline in both dementias that were of superior clinical plausibility. This vindicated our decision to incorporate KDE mixture modeling within the event-based model framework, which was motivated by applications such as this — where the data distribution is known to be non-Gaussian.

References

[1] A. L. Young, N. P. Oxtoby, J. Huang, R. V. Marinescu, P. Daga, D. M. Cash, N. C. Fox, S. Ourselin, J. M. Schott, D. C. Alexander, Multiple Orderings of Events in Disease Progression, in: S. Ourselin, D. C. Alexander, C.-F. Westin, M. J. Cardoso (Eds.), Information Processing in Medical Imaging, Springer, 2015, pp. 711–722. doi:10.1007/978-3-319-19992-4_56.

[2] D. W. Scott, On optimal and data-based histograms, Biometrika (1979) 605–610. URL http://www.jstor.org/stable/2335182

[3] E. Jones, T. Oliphant, P. Peterson, others, SciPy: Open source scientific tools for Python, 2001, [Online; accessed 2017-01-06].
URL http://www.scipy.org/

[4] M. Lehmann, J. Barnes, G. R. Ridgway, N. S. Ryan, E. K. Warrington, S. J. Crutch, N. C. Fox, Global gray matter changes in posterior cortical atrophy: A

serial imaging study, Alzheimer's & Dementia 8 (6) (2012) 502 – 512. doi:10.1016/j.jalz.2011.09.225.

[5] H. M. Fonteijn, M. J. Clarkson, M. Modat, J. Barnes, M. Lehmann, S. Ourselin, N. C. Fox, D. C. Alexander, An Event-Based Disease Progression Model and Its Application to Familial Alzheimer's Disease, in: G. Székely, H. K. Hahn (Eds.), Information Processing in Medical Imaging, Vol. 22, Springer Berlin Heidelberg, Berlin, Heidelberg, 2011, pp. 748–759. doi:10.1007/978-3-642-22092-0_61.

[6] H. M. Fonteijn, M. Modat, M. J. Clarkson, J. Barnes, M. Lehmann, N. Z. Hobbs, R. I. Scahill, S. J. Tabrizi, S. Ourselin, N. C. Fox, D. C. Alexander, An event-based model for disease progression and its application in familial Alzheimer's disease and huntington's disease, NeuroImage 60 (3) (2012) 1880–1889. doi:10.1016/j.neuroimage.2012.01.062.

[7] A. L. Young, N. P. Oxtoby, P. Daga, D. M. Cash, N. C. Fox, S. Ourselin, J. M. Schott, D. C. Alexander, A data-driven model of biomarker changes in sporadic Alzheimer's disease, Brain 137 (9) (2014) 2564–2577. doi:10.1093/brain/awu176.
